# Rethinking Functional Outcome Measures: The Development of a Novel Upper Limb Token Transfer Test to Assess Basal Ganglia Dysfunction

**DOI:** 10.3389/fnins.2018.00366

**Published:** 2018-05-30

**Authors:** Susanne P. Clinch, Monica Busse, Mariah J. Lelos, Anne E. Rosser

**Affiliations:** ^1^School of Biosciences, Cardiff University, Cardiff, United Kingdom; ^2^Centre for Trials Research, Cardiff University, Cardiff, United Kingdom; ^3^School of Medicine, Cardiff University, Cardiff, United Kingdom

**Keywords:** basal ganglia, Huntington's disease, functional ability, outcome measure, dual task, upper limb

## Abstract

The basal ganglia are implicated in a wide range of motor, cognitive and behavioral activities required for normal function. This region is predominantly affected in Huntington's disease (HD), meaning that functional ability progressively worsens. However, functional outcome measures for HD, particularly those for the upper limb, are limited meaning there is an imperative for well-defined, quantitative measures. Here we describe the development and evaluation of the Moneybox test (MBT). This novel, functional upper limb assessment was developed in accordance with translational neuroscience and physiological principles for people with a broad disease manifestation, such as HD. Participants with HD (*n* = 64) and healthy controls (*n* = 21) performed the MBT, which required subjects to transfer tokens into a container in order of size (Baseline Transfer), value (Complex Transfer) with and without reciting the alphabet (Dual Transfer). Disease specific measures of motor, cognition, behavior, and function were collected. HD patients were grouped into disease stage, from which, discriminative and convergent validity was assessed using Analysis of Variance and Pearson's correlation respectively. Manifest HD participants were slower than pre-manifest and control participants, and achieved significantly lower MBT total scores. Performance in the Complex Transfer and Dual Transfer tasks were significantly different between pre-manifest and stage 1 HD. All MBT performance variables significantly correlated with routinely used measures of motor, cognition, behavior, and function. The MBT provides a valid, sensitive, and affordable functional outcome measure. Unlike current assessments, MBT performance significantly distinguished the subtle differences between the earliest disease stages of HD, which are the populations typically targeted in clinical trials.

## Introduction

The basal ganglia is a highly organized group of interconnected, functionally subdivided, subcortical nuclei. Damage to the cortico-basal ganglia-thalamo circuitry plays an important role in multiple neurological conditions (Reiner and Deng, 2010), such as Parkinson's and Huntington's disease (HD), with symptoms progressively effecting standards of living. Neurodegenerative diseases are a leading cause of death globally (Kochanek et al., 2014) with limited potential for therapeutics to slow progression or prevent onset. In light of impending clinical trials of disease modifying interventions, well-defined clinical endpoints, and relevant objective progression criteria will be essential to progress potential therapies to regulatory approval with relative efficiency. A major challenge to date is the reliance on patient reported outcomes for the assessment of function. Functional assessments are crucial to gain an understanding about how standards of living change a disease progresses, and also following an intervention.

Selecting relevant outcome measures that best match the trial hypothesis is fundamental. To this end, there is an urgent need to develop novel assessments with high ecological validity and that also appropriately reflect the underlying neuropathology and the subsequent structure-function relationships (Chaytor and Schmitter-Edgecombe, 2003; Stout et al., 2016). Furthermore, to reduce the burden on patients and to reliably assess the effectiveness of developing therapies, it is important that assessments used in clinical trials are reliable, valid, as short as can be reasonably managed (in order to limit the burden on patients), and reflect clinically meaningful changes (Iansek and Morris, 2013).

HD is an autosomal dominant, inherited neurodegenerative disorder with a prevalence of 6–13/100,000 in the general population. People with manifest HD suffer from complex disease symptoms, including progressive motor, cognitive, and behavioral impairments, leading to gradual loss of functional independence and progressive escalation of healthcare costs over a 15–30 year period (Jones et al., 2016). Neurodegeneration in HD is widespread, but primarily involves degeneration of the cortico-basal ganglia-thalamo circuitry (Tabrizi et al., 2009; Lanciego et al., 2012; Novak et al., 2015), from which the striatum takes the brunt of the pathological burden. This degeneration is evident over a decade before symptom onset (termed pre-manifest) (Tabrizi et al., 2012), and recognized as manifest when motor symptoms begin, as rated on the Unified Huntington's disease rating scale (UHDRS) total motor score (UHDRS-TMS). The UHDRS-TMS is one of six standardized UHDRS assessments that are used to determine the range of clinical features associated with HD, which include a motor, cognitive, functional capacity, behavioral, functional assessment, and an independence scale (Kieburtz, 1996). Although the current gold standard, many of the UHDRS scales are limited by their ordinal ratings. Furthermore, it is difficult to clearly relate scores that focus on disease impairment to how they affect activities of daily living. A relatively recent systematic review of outcome measures used in HD pharmacological trials highlighted reliance on clinical reported outcomes in the assessment of function in HD (Carlozzi et al., 2014). Many of these assessments require a clinician assessment of a patient's ability to perform within relatively disparate domains, such as making a meal and dressing.

We suggest that performance based assessments that relate to daily function and importantly include a focus on fine motor skills are critical to the sensitive and reliable assessment of function. However, whilst there is increasing recognition of the importance of upper limb and fine motor assessment in HD (Brown et al., 1993; Whishaw et al., 2002; Saft et al., 2003; van Vugt et al., 2007; Klein et al., 2011; Collins et al., 2015), there remains relatively limited clinical literature on the topic. The Perdue Peg test (Tiffin and Asher, 1948) is a well-established upper limb functional test that has been used in a variety of settings and conditions but to our knowledge in two HD studies. The first where people with HD (*n* = 6) were found to perform slower than healthy controls (*n* = 12) (Brown et al., 1993) and the second, a far larger scale study in which peg insertion was found to discriminate between manifest HD (*n* = 140) and controls (*n* = 57) or pre-manifest HD (*n* = 34) but not pre-manifest HD and controls (Saft et al., 2003). The 10 euro neuro test is a simple timed coin alignment test that was developed with the express view of assessing finger dexterity in HD (van Vugt et al., 2007). It was found to be reliable and to discriminate between late stage HD (*n* = 10) and healthy controls (*n* = 14). There was some correlation with CAG repeat score however two of the 10 HD patients were not able to complete the tasks suggesting it could be subjective to floor effects. More recently, the nut and bolt test applied in pre-manifest HD (*n* = 24) and manifest HD (*n* = 27) and controls (*n* = 32) was shown to be a useful measure of fine-motor coordination in HD (Collins et al., 2015). Impairments in performance were seen at all stages of HD and in pre-manifest HD (non-dominant hand only) that were correlated with disease burden scores.

Many activities of daily living require performing and synchronizing multiple tasks (i.e., “dual-“ or “multi-tasking”), which can be challenging as it requires dividing attention between each task that is performed. This can lead to performance deterioration in one or both tasks, which is exacerbated in people with a neurological disorder such as HD (Delval et al., 2008; McIsaac et al., 2015; Vaportzis et al., 2015). The type of tasks combined as well as the task complexity can also have an impact on performance. One study revealed that people with HD had greater difficulty performing a motor-cognitive dual task than motor-motor (Delval et al., 2008), suggesting the former may be more sensitive to the cortico-basal ganglia circuitry disrupted in HD.

The Moneybox test (MBT) was developed to to specifically target functions that involve the cortico-basal ganglia circuitry to quantitatively reflect the neurodegeneration in HD. The MBT incorporates three motor-cognitive items that increase in task difficulty, plus two baseline items which are performed as single tasks.

**Figure 1:**
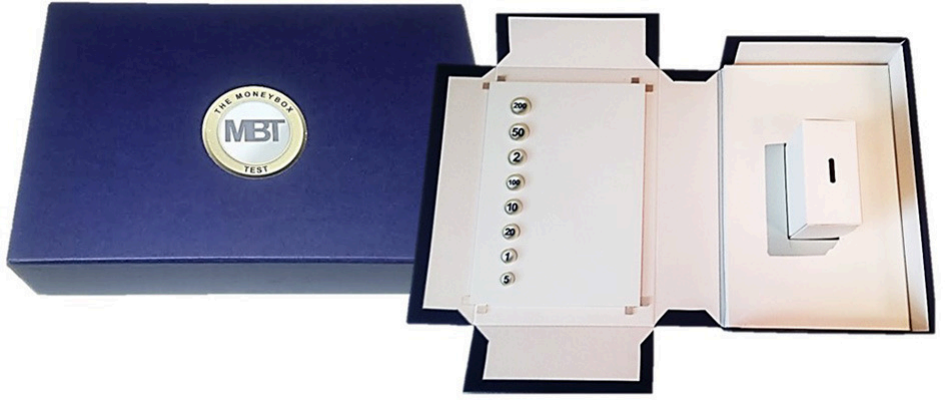
The Moneybox test is enclosed in a case when not in use and when opened the contents required for testing are revealed.

Here we report the development and validation of the Moneybox test (MBT; Figure 1). The aim of this study was to validate the MBT in people with all stages of HD and in a group of age matched, healthy controls. We hypothesized that people with advanced HD would perform more slowly and less accurately than those in the earlier disease stages and that all people with HD would perform more slowly and less accurately with increased item complexity.

## Materials and methods

### Participants

Eighty-five participants were recruited from the Cardiff HD research and management clinic between February 2016 and January 2017, from which 21 were gene-negative healthy controls (9 male) and 62 people were gene positive with HD (8 pre-manifest and 56 manifest; 38 male). The manifest group was further subdivided based on their UHDRS-Total functional capacity score (UHDRS-TFC) to form 4 groups (stage 1, TFC = 11–13 and TMS > 5; stage 2, TFC = 7–10; stage 3, TFC = 3–6; stage 4, 5 = 0–2). Participants in stages 4 and 5 were combined, forming the most advanced group. This was due to the lack of participants that were recruited in these stages. Ethical approval was obtained from South East Wales Research Ethics Committee (REC reference: 14/WA/1195). Inclusion criteria for the HD groups were (1) genetically confirmed diagnosis of HD; (2) over 18 years of age; (3) recruited onto Enroll-HD, which is a global observational study that provides researchers with access to non-identifiable clinical information (https://www.enroll-hd.org/). Exclusion criteria included an inability to provide informed consent and any comorbid condition that had the potential to confound the results of the study.

### Assessments

Specific criteria were established to guide the overall MBT development process (see Table 1).

**Table 1:** Criteria used to develop the MBT.

**Criteria**	**MBT**
Restricted to upper limb function	The MBT is performed seated and requires bilateral function to grasp, transfer and accurately release tokens into a container.
Ecologically valid	The MBT is a dual task assessment that consists of five items, from which three are transfer tasks with incremental difficulty; Baseline Transfer, Complex Transfer, and Dual Transfer tasks. The remaining two items are baseline tasks to ensure the subject can count backwards from values presented and recite the alphabet in preparation for the Complex Transfer and Dual tasks respectively. The MBT was designed so it was sensitive for individuals with different levels of functional ability, such as people with HD. Reciting the alphabet was used for the Dual Transfer task to increase task complexity. This specific task was selected as it is less likely to be confounded by education or job type compared to other commonly used secondary tasks, such as addition, subtraction, or verbal fluency tasks.
The assessment is applicable to people with all stages of HD	The MBT consists of a hierarchy of items with increasing levels of difficulty. Participants had to meet set criteria before proceeding to the more complex MBT items to minimize the chances of floor and ceiling effects. The pass/fail criteria is presented in the Supplementary Material.
The assessment is sensitive to functions that involve the degenerating neuroanatomy in HD	MBT items were developed to target behaviors that involve the cortico-basal ganglia-thalamo circuitry. This included: *Dexterity:* The lateral striatum is required for fine motor tasks (Döbrössy and Dunnett, 2003). To account for this, participants were required to pick up different sized tokens and accurately release these into a defined target on a container *Repeated motor transitions*: Rhythmic, repeated motor transitions leads to a change in neuronal firing patterns in the dorsolateral striatum (Ashby et al., 2010), and may relate to new skill learning (Turner and Desmurget, 2010). The MBT was designed to take advantage of these functions, as the participant is required to repeatedly transfer eight tokens as quickly as possible into a container *Oculomotor function* (Harting and Updyke, 2005): It was hypothesized that optimal MBT performance required occulo-motor function to rapidly saccade the eyes to the next token target *Attention* (De Diego-Balaguer et al., 2008)*:* The increasing levels of difficulty in the MBT intended to demand increasing levels of attention. Throughout the MBT, participants are required to transfer tokens between hands and in a given order. In the Dual Transfer task, attentional capacity is challenged again as participants are required to transfer tokens in a set order whilst simultaneously reciting the alphabet *Alphabet recitation:* Previous studies have shown that less cognitively demanding tasks can be more sensitive in people with HD than those with high cognitive demands (Snowden et al., 2001; Thompson et al., 2010). In addition, pre-clinical research suggests that the dorsolateral striatum is involved in performing fixed, automatic behaviors (Yin et al., 2004). Reciting the alphabet is a fairly simplistic task that is regularly recited from a young age. For many, by early adulthood, this recitation would pose little attentional demand as the memory is retrieved and automatically recited (Ashby et al., 2010; Turner and Desmurget, 2010). It was hypothesized that reciting the alphabet would load extra stress on the fronto-striatal circuitry making the Dual Transfer task more challenging for people with striatal dysfunction.
Minimal burden for the administrator and the participant	The MBT is uncomplicated to set up and takes between 5 and 10 min for the participant to perform. Due to the criteria developed for each MBT item, the length of the MBT assessment is dependent on the participant's functional ability. In addition, as the MBT is used to measure bilateral function, unlike pegboard tests, it only need to be performed once, which reduces the time of the assessment.
Compact	As clinic space is often limited and equipment needs to be stored and transported to different clinic locations, the MBT was designed so it was compact, lightweight and so construction involved few and small test components.
Quantitatively scored	The MBT is quantitatively evaluated, using time as a primary measure, which can be combined with accuracy to calculate an MBT total score. This method was used to improve inter-rater reliability and to sensitively measure change over time (Hobart et al., 2000).

**Figure 2:**
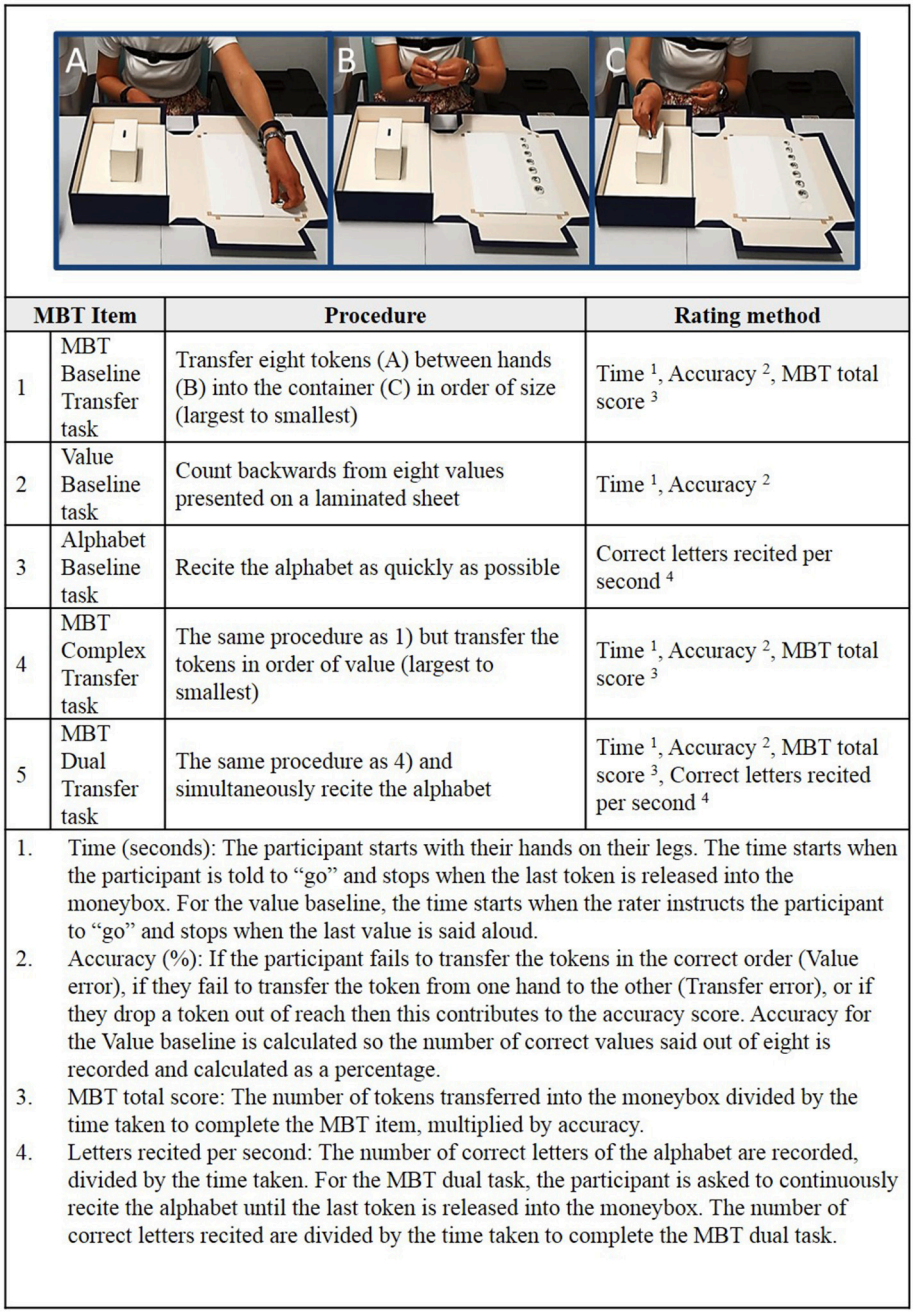
The Moneybox test (MBT) procedure and rating method. The subject is required to pick up the tokens with their non-dominant hand **(A)**, transfer to their dominant hand **(B)**, and release it into a moneybox **(C)**. The time (in seconds) taken to perform the transfer tasks, the accuracy (referring to any errors or dropped tokens made during the task) are recorded and used to calculate the MBT total score. The alphabet rate is the number of correct letters of the alphabet recited per second and used to compare alphabet baseline performance to alphabet performance during the Dual Transfer task.

The MBT procedure and the rating method is presented in Figure 2. The test items were carried out in the same order for each participant to ensure they could perform the baseline and the simpler tasks before proceeding to the more complex items. In addition, to minimize the floor, and ceiling effects, participants had to meet set criteria before proceeding to the more complex MBT items (described in Supplementary Material).

Additional information accessed through Enroll-HD included demographic information (age, gender and education level), the assessments from the UHDRS-TMS, TFC, functional score, independence score, and cognitive tests (Verbal fluency, Stroop task (word and color naming) and the Symbol digit modalities test) (Kieburtz, 1996). The CAG disease burden score was calculated [(CAGn – 35.5)^*^Age] to estimate how close pre-manifest participants were to developing manifest symptoms (Penney et al., 1997). The apathy and executive function summaries from the Problem behavior assessment, a questionnaire used to assess the behavioral symptoms in people with HD, were recorded (Craufurd et al., 2001). The physical and mental summary scores from the Short form-12 were used to evaluate correlations with health related quality of life (Ware et al., 1996). The Late-life functional disability instrument, a questionnaire used to understand how much difficulty the participant has performing common daily activities, was used to provide a measure of construct validity (Haley et al., 2002).

### Analyses

Demographic data and UHDRS scores were evaluated for all groups using the mean and the standard deviation. The mean performance scores were plotted with the standard error of the means (SEM) and the 95% confidence intervals.

#### Discriminative validity

Between group comparisons were made using analysis of variance (ANOVA). Independent subject factors included Group (control, pre-manifest or manifest participants) or TFC stage [TFC scores = 13 and UHDRS-TMS < 5, pre-manifest; 11–13, stage 1 (earliest symptomatic stage); 7–10, stage 2; 3–6, stage 3; 1–2, stage 4; and score of 0 is stage 5 (most advanced stage), or healthy controls]. Dependent subject factors included MBT time (time), and MBT total score (total), value time (value time), and number of correct letters said per second (alphabet rate). A two way repeated measures ANOVA was used to evaluate any change in performance within group with increased item complexity in the MBT Complex Transfer and MBT Dual Transfer task compared to the MBT Baseline Transfer. The MBT transfer items (Baseline Transfer, Complex Transfer, and Dual Transfer) and TFC group were used as factors. If the sphericity assumption was not met (*p* < 0.05), this was corrected using the Greenhouse-Geisser test. A Bonferroni *post-hoc* test was used for all ANOVA tests if results were deemed statistically significant (*p* < 0.05).

#### Convergent and construct validity

Pearson's correlation coefficients were used to reveal any associations between the MBT variables and the disease specific assessments of motor and cognitive ability, behavior, function and health related quality of life.

SPSS version 20 (PASW) (IBM Corporation, USA) was used for all analyses.

## Results

Demographic and clinical information for all participants are presented in Table 2. There was no significant difference in age between controls and HD gene positive subjects, however the pre-manifest HD group were significantly younger than the manifest participants [*F*_(5, 82)_ = 4.809, *p* < 0.05]. Those in stage 2 manifest HD were significantly older than healthy controls [*F*_(5, 79)_ = 3.285, *p* < 0.01]. Level of education was not significantly different between controls, manifest and pre-manifest participants [*F*_(2, 70)_ = 0.166, *p* = n.s.] or between any TFC disease stage [*F*_(5, 67)_ = 0.296, *p* = n.s.].

**Table 2:** Mean MBT participant demographic and clinical information revealed manifest subjects were significantly older than pre-manifest subjects, and stage 2 subjects were significantly older than healthy controls.

	**Controls**	**Pre-manifest**	**Manifest**	**Stage 1**	**Stage 2**	**Stage 3**	**Stages 4 and 5**
*N* (male: female)	21 (9:12)	8 (6:2)	56 (32:24)	23 (14:9)	17 (8:10)	11 (7:4)	3 (3:1)
Age	45.52 (15.03)	37.75 (6.43)	51.21 (12.19)	48.43 (9.01)	56.83 (15.69)	50.55 (10.63)	43.75 (5.68)
Total motor score		0.375 (0.52)	35.35 (21.07)	21.18 (12.1)	34.17 (12.24)	54.82 (19.23)	75 (20.88)
Total functional capacity		12.625 (0.52)	8.87 (3.64)	12.09 (0.92)	8.94 (1.16)	4.64 (1.36)	0.33 (0.58)
Functional scale		24.875 (0.35)	20.04 (5.97)	24.5 (0.74)	20.39 (1.46)	13.44 (4.5)	5 (7)
Independence scale		99.375 (1.77)	81.3 (13.77)	91.90 (7.49)	79.44 (6.62)	67.22 (7.55)	50 (28.28)
CAG disease burden score		260.71 (69.24)	400.29 (102.44)	373.39 (99.25)	411.78 (117.67)	424.73 (87.11)	429.38 (82.18)
Education level^†^	3.58 (1.51)	3.75 (0.89)	3.53 (0.91)	3.71 (1.06)	3.39 (0.78)	3.45 (0.69)	3.33 (1.53)

There were no significant differences between any other group for any other variable.

† *Education level was missing in n = 9 healthy controls*.

**Figure 3:**
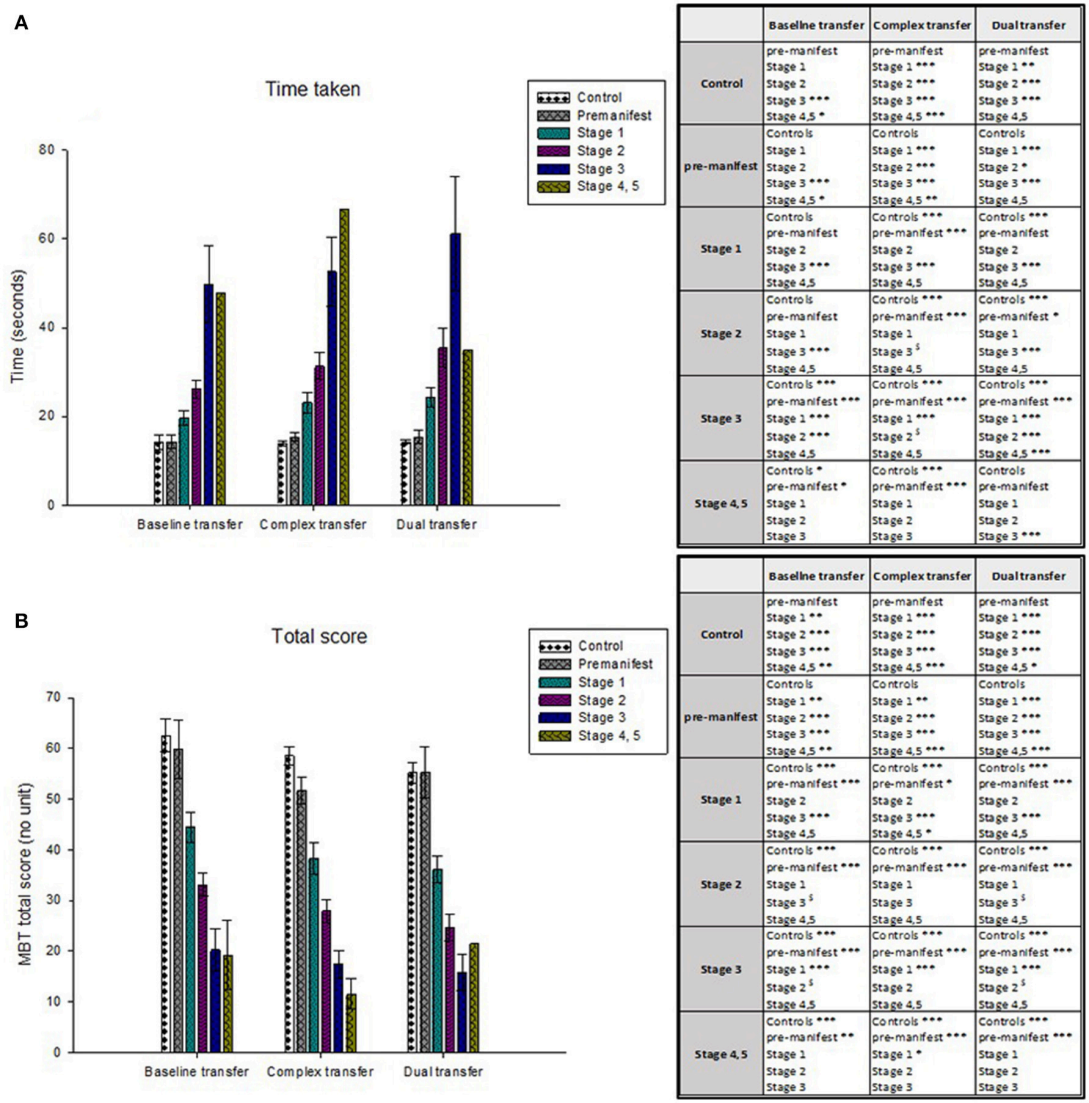
The mean time taken is plotted in **(A)** and the mean MBT total scores are plotted in **(B)**. Healthy controls and people in the early disease stages (stage 1) performed the transfer tasks significantly faster than those in the later disease stages (stage 4, 5). The same stepwise performance deterioration is evident according to the MBT total scores, from which healthy controls and people in the early disease stages achieved a greater MBT total score (indicative of a faster time and greater accuracy) compared to those in the more advanced disease stages. Significant differences between participant groups are presented in the tables, where ^*^p < 0.05, ^**^p < 0.01, ^***^p < 0.001.

Manifest participants were significantly slower in their performance of the MBT transfer tasks and achieved a significantly lower total scores than both pre-manifest and control participants. Performance in the MBT was also sensitive to disease stage. Participants in the more advanced disease stages were significantly slower and achieved lower MBT total scores than those in earlier disease stages (see Table 3 and Figure 3).

**Table 3:** Mean, standard deviation and 95% confidence intervals for time taken and mean MBT total scores achieved for each group (Control, Pre-manifest, Manifest; HD disease stage) during each of the MBT transfer items (MBT Baseline Transfer, Complex Transfer, and Dual transfer).

	**Group**	**Time taken (seconds)**	**95% confidence difference (upper-lower bound)**	**MBT total (no unit)**	**95% confidence difference (upper-lower bound)**
**MBT Baseline Transfer**	Control	14.34 ± 2.41	9.54 − 19.14	62 ± 2.73	56.55 − 67.45
	Pre-manifest	13.33 ± 4.46	4.45 − 22.21	62.69 ± 5.06	52.61 − 72.78
	Manifest	27.85 ± 2.15	23.301 − 32.15	34.97 ± 1.85	31.05 − 38.68
	ANOVA: F value and p value	*F*_(2, 80)_ = 9.551, *p* < 0.001		*F*_(2, 80)_ = 35.178, *p* < 0.001	
	Stage 1	20.59 ± 2.33	15.95–25.24	42.2 ± 2.65	36.92–47.47
	Stage 2	23.8 ± 2.65	18.51–29.08	35.18 ± 3.01	29.18–41.19
	Stage 3	46.62 ± 3.42	39.8–53.44	20.4 ± 3.89	12.65–28.14
	Stage 4, 5	37.85 ± 6.3	25.29–50.41	27.77 ± 7.16	13.5–42.04
	ANOVA: *F*-value and *p*-value	*F*_(5, 72)_ = 14.25; *p* < 0.001		*F*_(5, 72)_ = 21.44; *p* < 0.001	
**MBT Complex Transfer**	Control	13.72 ± 2.39	8.95–18.49	59.32 ± 2.11	55.1–63.54
	Pre-manifest	14.78 ± 4.34	6.12–23.44	52.88 ± 3.84	45.22–60.55
	Manifest	33.59 ± 2.43	28.25–38.28	28.49 ± 1.73	25.06–32.23
	ANOVA: *F*-value and *p*-value	*F*_(2, 75)_ = 17.395, *p* < 0.001		*F*_(2, 75)_ = 61.856, *p* < 0.001	
	Stage 1	24.15 ± 2.39	19.38–28.91	36.51 ± 2.11	32.29–40.73
	Stage 2	30.4 ± 2.68	25.05–35.74	22.51 ± 2.37	22.51–31.97
	Stage 3	51.51 ± 3.67	44.19–58.83	17.05 ± 3.25	10.54–23.53
	Stage 4, 5	54.64 ± 6.14	42.39–66.88	16.37 ± 5.43	5.53–27.21
	ANOVA: *F*-value and *p*-value	*F*_(5, 67)_ = 20.78; *p* < 0.001		*F*_(5, 67)_ = 38.45; *p* < 0.001	
**MBT Dual Transfer**	Control	14.14 ± 2.13	9.89–18.39	55.67 ± 2.12	51.44–59.9
	Pre-manifest	14.89 ± 3.86	7.17–22.61	56.03 ± 3.84	48.35–63.71
	Manifest	33.44 ± 2.69	27.45–38.62	28.2 ± 1.64	25.05–31.87
	ANOVA: *F*-value and *p*-value	*F*_(2, 71)_ = 14.745, *p* < 0.001		*F*_(2, 71)_ = 55.864, *p* < 0.001	
	Stage 1	25.01 ± 2.13	20.76–29.26	34.75 ± 2.12	30.53–38.98
	Stage 2	28.97 ± 2.45	24.08–33.87	27.32 ± 6.66	22.45–32.19
	Stage 3	65.58 ± 3.86	57.86–73.3	13.58 ± 3.84	5.9–21.26
	Stage 4,5	32.44 ± 6.69	19.07–45.81	22.43 ± 6.66	9.12–35.73
	ANOVA: *F*-value and *p*-value	*F*_(5, 63)_ = 29.21; *p* < 0.001		*F*_(5, 68)_ = 31.38; *p* < 0.001	

Performance in the control and pre-manifest groups did not differ with increased task complexity. Participants across manifest disease stages were however significantly slower during performance of the more complex items (Complex Transfer and Dual Transfer task), compared to the Baseline Transfer [TFC stage x MBT item: Baseline Transfer vs. Complex Transfer, *F*_(5, 72)_ = 4.65, *p* < 0.001; Baseline Transfer vs. Dual Transfer, *F*_(5, 68)_ = 7.68, *p* < 0.001; Complex Transfer vs. Dual Transfer, *F*_(5, 68)_ = 14.27, *p* < 0.001]. In addition, more participants in the advanced disease stages failed to meet the pass/fail criteria required to proceed to the Complex Transfer and Dual Transfer items. This resulted in smaller group sizes as MBT items increased complexity and reduced the stage 4, 5 group from 100 to 33% (from *n* = 3 to *n* = 1) in the Dual Transfer task. In comparison, 95 and 100% of the control and pre-manifest participants completed the whole MBT assessment (*n* = 20 and *n* = 8 respectively).

Performance for all MBT variables significantly correlated with UHDRS measures except for the rate the alphabet was recited in the MBT Dual Transfer task vs. the verbal fluency and the Stroop color naming (see Table 4). Performance scores in the MBT items significantly correlated with CAG disease burden score, as well as the current performance based functional measures used for Enroll-HD (Timed up and go, and sit to stand), and the Late-Life Functional Disability Instrument. MBT items also correlated with the SF-12 physical summary, but not the SF-12 mental summary or the executive function score from the Problem behavior assessment. The Dual Transfer total score also significantly correlated with the apathy calculation from the Problem behavior assessment.

**Table 4:** MBT convergent validity revealed that MBT performance (time and MBT total score) in the transfer tasks significantly correlated with all UHDRS motor, function, and cognitive assessments.

	**UHDRS–TMS**	**UHDRS–TFC**	**TFC stage**	**UHDRS-FAS**	**UHDRS iIndependence scale**	**UHDRS symbol digit correct**	**UHDRS verbal fluency correct**	**UHDRS Stroop color-name correct**	**UHDRS Stroop word-reading correct**	**CAG disease burden score**	**Timed Up and Go**	**Sit to stand**	**PBA apathy**	**PBA executive function**	**SF-12 physical summary**	**SF-12 mental summary**	**LL-FDI total**	**LLFDI-upper extremity**	**LLFDI-lower extremity**
MBT Time Baseline Transfer	0.775^**^	−0.627^**^	0.621^**^	−0.673^**^	−0.580^**^	−0.565^**^	−0.466^**^	−0.591^**^	−0.589^**^	0.379^**^	0.953^**^	−0.653^**^	0.1	−0.07	−0.396^*^	0.112	−0.423^**^	−0.460^**^	−0.437^**^
MBT Time Complex Transfer	0.812^**^	−0.718^**^	0.719^**^	−0.702^**^	−0.651^**^	−0.619^**^	−0.480^**^	−0.608^**^	−0.633^**^	0.411^**^	0.915^**^	−0.700^**^	0.183	0.093	−0.351^*^	0.039	−0.439^**^	−0.448^**^	−0.471^**^
MBT Time Complex Transfer	0.732^**^	−0.678^**^	0.632^**^	−0.678^**^	−0.635^**^	−0.559^**^	−0.391^**^	−0.563^**^	−0.587^**^	0.237	0.940^**^	−0.702^**^	0.174	0.113	−0.399^*^	−0.284	−0.517^**^	−0.537^**^	−0.537^**^
MBT Total Baseline Transfer	−0.756^**^	0.633^**^	−0.618^**^	0.584^**^	0.574^**^	0.716^**^	0.468^**^	0.631^**^	0.609^**^	−0.489^**^	−0.667^**^	0.501^**^	−0.183	−0.041	0.355^*^	0.109	0.507^**^	0.482^**^	0.468^**^
MBT Total Complex Transfer	−0.791^**^	0.682^**^	−0.678^**^	0.616^**^	0.639^**^	0.719^**^	0.432^**^	0.599^**^	0.582^**^	−0.487^**^	−0.649^**^	0.560^**^	−0.242	−0.147	0.345^*^	0.098	0.475^**^	0.413^**^	0.441^**^
MBT Total Dual Transfer Task	−0.777^**^	0.655^**^	−0.604^**^	0.586^**^	0.674^**^	0.741^**^	0.428^**^	0.647^**^	0.633^**^	−0.377^**^	−0.637^**^	0.570^**^	−0.294^*^	−0.155	0.326	0.257	0.488^**^	0.438^**^	0.461^**^
Value Baseline	0.674^**^	−0.631^**^	0.639^**^	−0.701^**^	−0.666^**^	−0.546^**^	−0.432^**^	−0.581^**^	−0.609^**^	0.302^*^	0.704^**^	−0.561^**^	0.392^**^	0.156	−0.447^**^	0.081	−0.433^**^	−0.513^**^	−0.456^**^
Alphabet Baseline	−0.439^**^	0.475^**^	−0.367^**^	0.462^**^	0.421^**^	0.421^**^	0.348^**^	0.415^**^	0.400^**^	−0.185	−0.197	0.535^**^	−0.089	−0.254^*^	0.393^*^	0.266	0.422^**^	0.453^**^	0.462^**^
Alphabet Dual Task	−0.405^**^	0.419^**^	−0.388^**^	0.312^*^	0.411^**^	0.428^**^	0.113	0.267	0.316^*^	−0.202	−0.338	0.418^*^	−0.365^**^	−0.123	0.356^*^	0.28	0.567^**^	0.444^**^	0.497^**^

Performance in all transfer tasks significantly correlated with the SF-12 physical summary and functional assessments including the Timed Up and Go, Sit to Stand and the Late Life Functional Disability Instrument domains. There was also a significant correlation between the Dual Transfer and Problem Behavior Assessment Apathy score. MBT, Moneybox test; UHDRS, Unified Huntington's disease rating scale; TMS, Total motor score; TFC, Total functional capacity; FAS, functional assessment score; PBA, Performance behavior assessment; SF-12, Short-form 12; LL-FDI, Late life functional disability questionnaire.

* p < 0.05 (light gray);

** *p < 0.01 (dark gray)*.

## Discussion

The MBT was developed with the express aim of supplying clinicians and researchers with a functional upper limb assessment that is sensitive to people with all stages with HD and therefore reflects the progressive basal ganglia degeneration in this disease. Performance in the MBT could distinguish between people gene positive with HD and healthy controls, as well as people with different stages of HD. Participants with manifest HD performed significantly more slowly and less accurately with increasing item complexity, resulting in a lower MBT total score compared to that seen in pre-manifest and control groups. MBT performance also significantly correlated with the UHDRS, quality of life, and functional questionnaire measures.

The mean MBT total scores revealed that the MBT was sensitive to all stages of disease, but not between controls and pre-manifest HD participants. This could have been due to the relatively small pre-manifest sample (*n* = 8). Overall, the time taken to perform the MBT and the MBT total score deteriorated in a stepwise manner between groups as HD progressed. Control and pre-manifest participants performed the MBT most quickly and achieved the greatest total scores, whereas stage 4 and 5 participants were slowest in their performance of the MBT. We believe that the successful performance of the MBT requires intact basal ganglia function given the complex motor planning, motor initiation and motor accuracy required in the test (Turner and Desmurget, 2010; Dudman and Krakauer, 2016). Thus, as the cortico-basal ganglia-thalamo circuitry progressively degenerates in people with HD (Despard et al., 2015), this could lead to slower and less accurate MBT performance, resulting in lower MBT total scores.

One reason for slower performance in more advanced disease stages could be increased difficulty automating tasks. In a previous study, healthy controls, and people with pre-manifest HD gradually improved in a motor skill task when repeatedly performed, whereas people with manifest HD did not (Shabbott et al., 2013). Participants were required to use their finger to direct a cursor to a target that was reflected onto a mirror. The results from the study revealed that people with manifest HD were slower, less accurate and produced more variable trajectories over repeated sessions, whereas controls and pre-manifest participants improved, gradually becoming quicker whilst remaining accurate over sessions. One reason for this could be that controls and pre-manifest HD participants have the ability to automate movement, which in turn would free attentional resources that could be directed to the secondary task (Thompson et al., 2010). Given that the basal ganglia is implicated in automatic, habitual tasks, it could be involved in automating aspects of dual task performance (Saling and Phillips, 2007; Ashby et al., 2010; Kim and Hikosaka, 2015). Therefore, it may be that with increasing basal ganglia circuit degeneration the difficulties in multi-tasking experienced by people with HD are two-fold; Not only do they have limited attentional capacity, but they may also have difficulty carrying out simple, automatic tasks.

**Figure 4:**
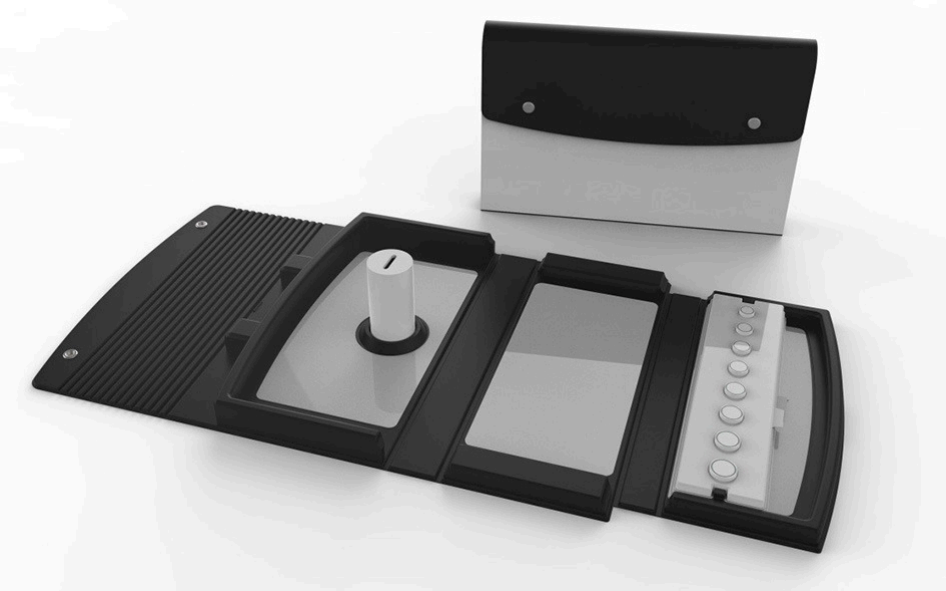
The Clinch Token Transfer Test (C3t) is an optimized version of the Moneybox test (MBT). In preparation for dissemination and use in clinical trials, the C3t was developed so test components are made of robust materials that can be cleaned. It is also compact, with all test components contained within the case. The test procedure is more efficient, with token trays prepared and stacked ready for use so there is no set up time for the researcher between assessment items. Although the C3t procedure is ultimately the same as the MBT, unlike the MBT, the Dual Transfer task consists of tokens with different values to those in the Complex Transfer, which was added to reduce potential practice effects. Therefore, in the C3t, an additional value baseline item was added (Complex Value baseline), which proceeds the original Simple Value baseline.

Importantly, participant performance in pre-manifest and stage 1 groups significantly differ for the Complex Transfer and Dual Transfer tasks; these disease stages being the most commonly targeted for clinical trials to test the effectiveness of new treatments (Glorioso et al., 2015; Kumar et al., 2015). To our knowledge, there is no other functional upper limb assessment available that is able to distinguish the subtle performance differences between pre-manifest HD and stage 1. This difference was only evident in the more complex MBT items (Complex Transfer and Dual Transfer tasks) and not in the Baseline Transfer, which supports our approach of incorporating different levels of complexity when assessing functional ability in HD. An initial hypothesis was that participant performance would deteriorate with increased item complexity. Although participants in stages 1, 2, 3, and 4, 5 performed significantly more slowly in the Complex Transfer and Dual Transfer tasks relative to baseline, there was no significant performance difference between the Complex Transfer and Dual Transfer task in stages 1, 2, or 3. One explanation for this could be practice effects due to the familiarity of the values presented on the tokens. To overcome this, a new version of the MBT has been developed entitled the Clinch token transfer test (C3t; see Figure 4). This consists of tokens with different values for the Complex Transfer and Dual Transfer task. As healthy controls and pre-manifest participants maintained performance across the MBT Baseline Transfer, Complex Transfer and Dual Transfer task, the aim of the Clinch token transfer test is to reduce the chances of practice effects and to test whether performance differs with increased complexity between healthy controls and people with pre-manifest HD.

Uniquely in this study, we have considered relationships between performance on the MBT and relevant clinical domains. Performance scores (time taken and MBT total) in the MBT Transfer items significantly correlated with all UHDRS measures, evidencing strong convergent validity. This was also true for the SF-12 physical summary, the function component of the Late-Life Functional Disability Instrument, the Timed up and Go and the Sit to stand, which are all measures used to assess performance in daily functional tasks. MBT transfer tasks were also correlated with CAG disease burden score, which again suggests that MBT performance is capable of tracking disease stage in HD. The CAG disease burden score is particularly useful for pre-manifest patients as they are typically heterogeneous, with some people closer to disease onset than others (Klöppel et al., 2015). As the pre-manifest group in this study was relatively small (*n* = 8), an aim for future research involves recruiting a larger group of pre-manifest patients to identify if the MBT is capable of identifying people far from and close to manifest disease onset. Interestingly the MBT Dual Transfer total also correlated with the apathy score from the Problem behavior assessment, revealing that the more apathetic the subject, the worse the total score in the MBT Dual Transfer task. This suggests that more complex tasks could be helpful identifying apathetic from non-apathetic subjects. However, we suggest other apathy assessments such as the Apathy Evaluation scale (Clarke et al., 2011) would also need to be used to provide a reliable conclusion to these findings. The MBT did not correlate with the executive function summary from the Problem behavior assessment. However, as the MBT items significantly correlated with the Symbol digit test, the Stroop tasks and the Letter verbal fluency, which are measures of executive function (Craufurd and Snowden, 2002), this suggests that the ordinal scale used to rate the Problem behavior assessment may not provide as accurate a measure of executive function as performance based measures do.

Additionally, results also revealed that all MBT items significantly correlated with the upper limb score of the Late-Life Functional Disability Instrument, suggesting that MBT performance relates to daily tasks that require upper limb function. Furthermore, all MBT scores also correlated with the lower limb score of the Late-Life Functional Disability Instrument. This suggests the MBT could be used as a general measure of function in HD.

The MBT is a novel dual-task assessment that has potential to provide sensitive feedback to clinicians and researchers regarding upper limb function in people with HD. The objective scoring methods that are used in the MBT, as opposed to ordinal rating scales, are crucial for interventions, such as cell transplantation, where symptom changes are gradual and can be subtle (Wijeyekoon and Barker, 2011). As an assessment, the MBT is quick to perform, inexpensive to produce and easily stored, which avoids the common problem of restricted space in clinical settings. Furthermore, the fact that it requires minimal researcher training and is independent of both language and culture barriers makes it an attractive outcome measure for clinical trials globally. The test can also be supplemented with accelerometers for the in-depth assessment of motion parameters. These have been used in previous studies to measure gait parameters in people with pre-manifest and manifest HD, and to quantify tremor severity in people with Parkinson's disease (Patel et al., 2012; Dalton et al., 2013). We have applied them in HD during the performance of the MBT, and were able to identify movement features that were able to distinguish between manifest and pre-manifest HD groups (Bennasar et al., 2016). Subsequent to the development of the initial MBT, the test has undergone some minor amendments to develop an advanced prototype for full evaluation in a clinical setting. It has been renamed as Clinch Token Transfer Test (C3t).

A limitation of the MBT is the increased exposure to the token values across the value baseline, Complex Transfer and Dual Task. To minimize the chances of practice effects, the C3t test procedure was designed so different token values are presented for the Transfer Complex and the Dual task. Furthermore, as a caveat of this study is the lack of longitudinal data, future work will focus on evaluating performance over time of the new version of the MBT (namely, the C3t), as well as extending the application to other conditions with basal ganglia dysfunction, such as Parkinson's disease and subtypes of epilepsy.

## Author contributions

The authors made substantial contributions to the conception, acquisition, analysis, and interpretation of data for this study. SC and MB developed the Moneybox test, collected and analyzed data and wrote the first draft of the manuscript. MB critically reviewed all aspects of the study. All authors gave their final approval of the version to be published and agreed to be accountable for all aspects of the work.

### Conflict of interest statement

The authors declare that the research was conducted in the absence of any commercial or financial relationships that could be construed as a potential conflict of interest.
