# Supplementary material for: Rethinking Functional Outcome Measures: The Development of a Novel Upper Limb Token Transfer Test to Assess Basal Ganglia Dysfunction
Source: Front Neurosci. 2018 May 30;12:366. doi: 10.3389/fnins.2018.00366 (PMC5988893; doi:10.3389/fnins.2018.00366)
Supplement: Supplementary file 1 [file Image_1.PDF]

Supplementary Material

Rethinking functional outcome measures: The development of an upper limb test to assess basal ganglia dysfunction

Susanne P. Clinch PhD, Monica Busse PhD, * Mariah J. Lelos PhD, and Anne E. Rosser PhD FRCP

*** Correspondence:**

Monica Busse
Centre for Trials Research
Neuadd Meirionnydd, Heath Park
Cardiff University,
Wales, UK
E-mail: busseme@cardiff.ac.uk

1 Supplementary Figures and Tables

To minimize floor and ceiling effects, set criteria was developed for each MBT item. The subject was required to pass the criteria to continue to the next, more complex phase of the assessment. If the subject failed to pass one or more of the criteria then the subject was stopped and they did not continue to the next assessment item.

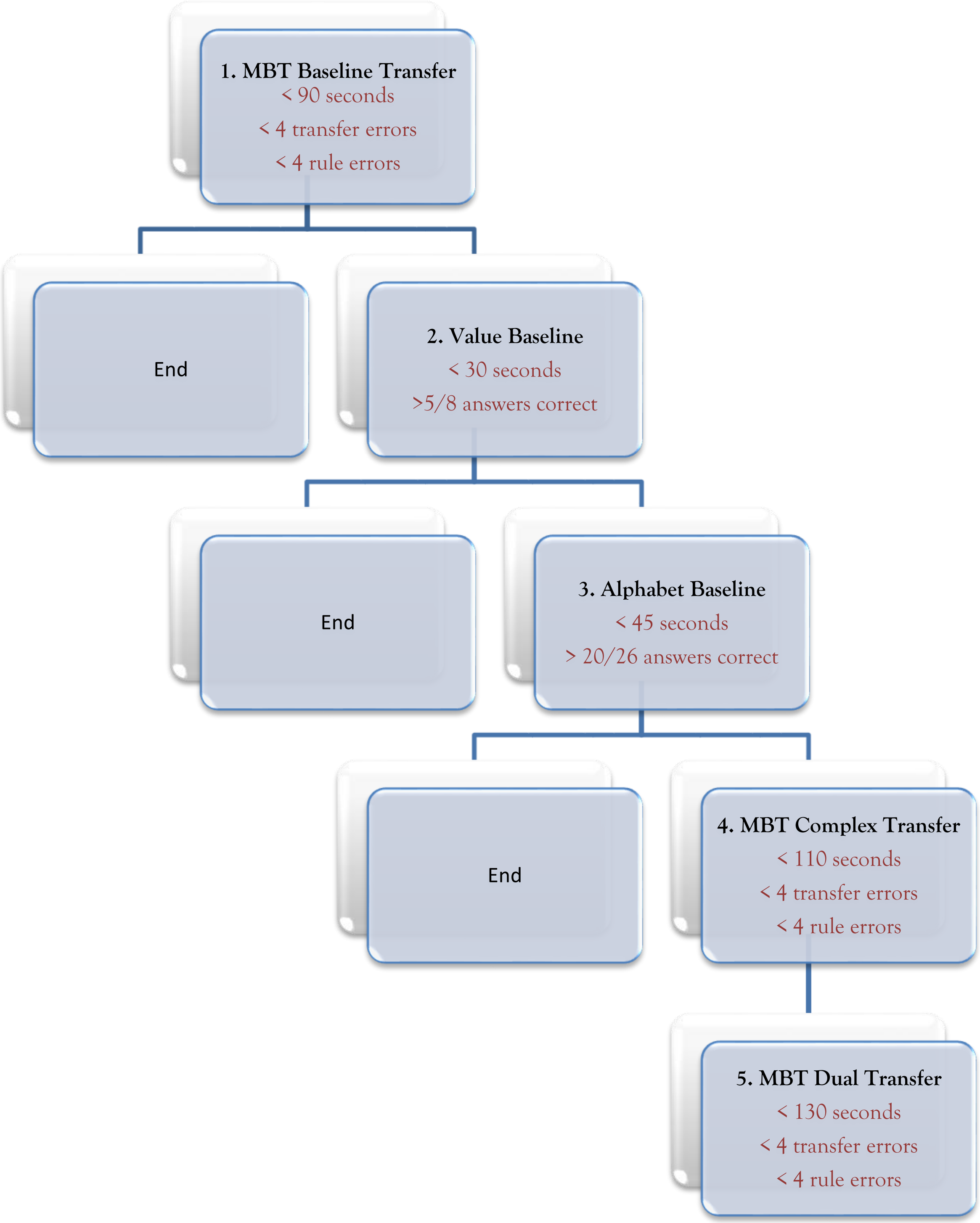

Figure 1: The pass/fail criteria used for the MBT assessment. Text in red describes the set criteria required to pass the next phase of the assessment. If the subject did not meet the criteria, the MBT ended there. Where MBT = Moneybox test.
